# HOW SKIN TONE INFLUENCES RELATIONSHIPS BETWEEN DISCRIMINATION AND MENTAL HEALTH AMONG OLDER AFRICAN AMERICANS

**DOI:** 10.1093/geroni/igad104.0441

**Published:** 2023-12-21

**Authors:** Tyrone Hamler

**Affiliations:** University of Denver, Denver, Colorado, United States

## Abstract

Skin tone has been identified as an important factor for within-group health disparities. We examine the moderating role of skin tone in the relationship between discrimination, self-rated mental health, and serious psychological distress (SPD) and (b) whether moderated by gender. We used a subsample of African Americans aged 55+ (N = 837) from the National Survey of American Life. Mental health outcomes were SPD and self-rated mental health. Results indicate discrimination was associated with worse self-rated mental health and SPD in the total sample and among women. Skin tone moderated the association between discrimination and SPD among both genders. The effect of discrimination on mental health was stronger among darker-skinned respondents than lighter respondents. Gender-stratified analyses indicated skin tone moderated the association between discrimination and self-rated mental health for men but not women. The negative psychological effects associated with darker complexion indicate one mechanism linking discrimination and mental health.

